# Secure Exchange of Digital Metrological Data in a Smart Overhead Crane

**DOI:** 10.3390/s22041548

**Published:** 2022-02-17

**Authors:** Tuukka Mustapää, Henri Tunkkari, Jaan Taponen, Leo Immonen, Wiebke Heeren, Oksana Baer, Clifford Brown, Raine Viitala

**Affiliations:** 1 Department of Mechanical Engineering, School of Engineering, Aalto University, 02150 Espoo, Finland; henri.tunkkari@aalto.fi (H.T.); jaan.taponen@aalto.fi (J.T.); leo.immonen@aalto.fi (L.I.); raine.viitala@aalto.fi (R.V.); 2 Division 1 Mechanics & Acoustics, Physikalisch-Technische Bundesanstallt, 38116 Braunschweig, Germany; wiebke.heeren@ptb.de (W.H.); oksana.baer@ptb.de (O.B.); clifford.brown@ptb.de (C.B.)

**Keywords:** data trustworthiness, digital SI, digital calibration certificate, IoT communication, metrology, traceability

## Abstract

Digitalization and the rapid development of IoT systems has posed challenges for metrology because it has been comparatively slow in adapting to the new demands. That is why the digital transformation of metrology has become a key research and development topic all over the world including the development of machine-readable formats for digital SI (D-SI) and digital calibration certificates (DCCs). In this paper, we present a method for using these digital formats for metrological data to enhance the trustworthiness of data and propose how to use digital signatures and distributed ledger technology (DLT) alongside DCCs and D-SI to ensure integrity, authenticity, and non-repudiation of measurement data and DCCs. The implementation of these technologies in industrial applications is demonstrated with a use case of data exchange in a smart overhead crane. The presented system was tested and validated in providing security against data tampering attacks.

## 1. Introduction

Digitalization and the growth of the Internet of Things (IoT) has led to vast amounts of data being collected in all kinds of settings. The availability of data and advanced data analyzing methods, e.g., machine learning, have made it possible to pursue efficiency, sustainability, and safety in the different sectors of the society [1,2]. Typical application and development areas have been, e.g., smart cities [3,4], smart grids [5], and smart logistics [6,7]. A significant part of the IoT systems are linked to industrial applications, which is referred to as the Industrial IoT (IIoT) or Industry 4.0, where data are used to optimize manufacturing processes [8], decision-making and management [6,9], condition monitoring and predictive maintenance [10], and many other purposes [11].

However, even though these applications are heavily dependent on data, in many IoT applications, the quality and trustworthiness of the data collected by individual sensors remain unknown or vague. This sets limits for the potential usefulness of the data. These problems are commonly addressed with the use of different types of post-processing methods [12,13]. In addition, IoT solutions have typically been based on at least partially closed systems where the data collected have been stored and used more or less locally, e.g., at manufacturing facilities. As the data have not been transferred to external systems, the need for more advanced data formats or metadata has not been essential for system operation. The need for cost efficiency and development towards more open communication and data exchange between parties have set more demands for the systems to achieve full interoperability, which is why community-based development and standardizing of technologies have become common in the world of the IIoT [14].

Traditionally, the measurement of data trustworthiness has been studied in metrology, which is the science concentrating on measurements and the establishment of the common understanding of units representing physical quantities and maintaining the corresponding infrastructure. Due to the complexity of the metrology infrastructure and the variances in the practices in different domains, the digitalization of metrology has not been as fast as in most industrial areas [15,16]. Thus, it has become somewhat of a limiting factor in the digitalization of industrial quality management processes. The digitalization of metrology pursues offering the means for the correct interpretation of the data and having the important metadata, such as measurement uncertainty and traceability information, available for use in automated IoT systems, e.g., via digitized data formats offering the capability for machine reading and processing of these data [12,13,17,18]. In this paper, we refer to these data or metadata for presenting the metrological information in a machine-readable and interpretable format as digital metrological data. The ongoing work includes the introduction of the formats for digital calibration certificates (DCCs) [19,20,21,22] and digital SI (D-SI) [23], which are being developed to provide the basis for the universal exchange of metrological data.

In IoT systems where, e.g., heterogeneous sensor networks are used, one of the key requirements for the functionality of the systems is the interoperability of the interfaces and data formats in which the data are being transmitted by the devices and software [12]. Thus, also standardization is essential for the development of cost-efficient IoT systems. Another important aspect of ensuring data trustworthiness is the ability to validate the traceability of the data for which the authenticity and integrity of the data come into question. Examples of industries that have a particular interest in the trustworthiness of measurement data integrity and trustworthiness include, e.g., the pharmaceutical industry (due to patient safety) and the car industry (vast global subcontracting networks). One of the domains facing this issue is logistics and cargo handling, where trustful data are required for tracking the goods and containers to ensure both their security and origin, e.g., in the case of reducing the use of conflict minerals [24].

Millions of tons of goods are transported daily via containers loaded and unloaded in harbors. From 2016, the Safety of Life at Sea (SOLAS) convention of the International Maritime Organization (IMO), which regulates the minimum safety standards related to the construction, equipment, and operation of merchant ships, requires that the weight of the containers must be shared with carriers [25]. The reasoning behind this decision was due to the fact that knowing the weights of individual containers is essential for determining the weight distribution and thus the stability of the carrier ship. If these data are not accurate, the ship may capsize due to poor stability, as was the case presented in [26]. Currently, the systems and methods for collecting and presenting these data can vary greatly between applications as there are great differences, e.g., in the capabilities in adapting to the use of IoT systems that enable integrating the measuring systems into the cargo-handling systems or cranes. In most IoT applications, the measurement data do not include any metadata about the used measurement instruments and their measurement uncertainty and traceability, which are considered essential in metrology. Without the metadata, it is impossible to assess the consistency and comparability of measurements conducted in different locations, e.g., harbors where containers are weighed.

One of the research projects covering the digitalization of metrology is the EMPIR Project 17IND02 SmartCom funded through the European Union’s Horizon 2020 Programme. The central mission of the SmartCom project is to develop and provide the basis for a secure, unambiguous, and unified exchange of data in all communication networks where metrological data are used [27,28]. To test and validate the research outcomes of SmartCom in industrial end-user applications, two demonstrators were developed as a part of the project [29]. The demonstrator presented in this paper showcases the use of DCCs, D-SI, and appropriate cryptographical methods for the secure exchange of the measurement data and relevant metadata of cargo containers.

In this paper, we report the following original contributions:

1. We present a method for how digital metrological data as metadata can be used to enhance the trustworthiness IoT data;
2. We propose how to use data security technologies and cryptographical methods alongside DCC and D-SI applications;
3. We introduce a demonstrator for integrating the digital data formats and necessary security technologies into IIoT systems with the use case being exchanging metrological data in a smart overhead crane similar to the ones that are used in harbors.

The paper is organized as follows: Section 2 provides the relevant background of the research activities in the digitalization of metrology, a brief insight into the digital signatures and distributed ledgers, and the description of the smart overhead crane that was used as the demonstrator platform. The demonstrator implementation is presented in Section 3, and the results and their validation are discussed in Section 4. The opportunities arising from the digitalization of metrology, remaining challenges, and research topics are discussed in Section 5. Section 6 concludes the paper.

## 2. Related Work

### 2.1. Current Practices and Standards in IIoT Communication

The vast number of device manufacturers, service providers, and eventual end-use applications has meant that the implementation of IoT systems has required much work to ensure the interoperability of the data formats, communication protocols, and interfaces. The need for the interoperability and cost efficiency of IoT systems has led to the formation of development communities that are working on standardizing solutions covering entire production life-cycles in different domains. Examples of the resulting technologies and standards developed by these kinds of communities include:

- Open Platforms Communication Unified Architecture (OPC UA (https://opcfoundation.org/about/opc-technologies/opc-ua/, accessed on 30 August 2021)) by the OPC foundation;
- NAMUR Open Architecture (NOA (https://www.namur.net/en/focus-topics/namur-open-architecture/, accessed on 30 August 2021)) by the User Association of Automation Technology in Process Industries NAMUR;
- FOUNDATION Fieldbus (https://www.fieldcommgroup.org/technologies/foundation-fieldbus/foundation-technology-overview, accessed on 30 August 2021) by FieldComm Group;
- PROFIBUS (https://www.profibus.com/download/profibus-technology-and-application-system-description/, accessed on 30 August 2021) by PROFIBUS and PROFINET International (PI).

The actual data formats that are used can vary greatly based on the application. The formats can be unstructured, semi-structured, or structured, which means that the data can be very heterogeneous. The data from low-cost IoT sensors are often unstructured, which means that there are very few if any metadata included in the data samples describing the context of the measurement, which limits the value of the data as the data quality and trustworthiness of the data are largely unknown [13,30]. Unstructured data formats also quickly lead to interoperability issues. In applications where more structured data formats are needed, formats such as the Extensible Markup Language (XML) [31] or JavaScript Object Notation (JSON) [32] are commonly used. Typical communication protocols include the Hypertext Transfer Protocol (HTTP) [33] and Message Queuing Telemetry Transport (MQTT) [34].

In the IoT, as well as generic web applications, the interaction between the applications is implemented using Application Programming Interfaces (APIs). APIs enable efficient integration of different system or software modules while ensuring their interoperability. There exist numerous architecture styles that are commonly used for APIs. The most commonly used architecture type is Representational State Transfer (REST), which defines requirements for interface uniformity, client–server independence, statelessness, cacheability, the allowance of layered systems, and the availability of the executable code. An API must fulfill these to be considered as a RESTful API (https://restfulapi.net/, accessed on 10 September 2021) when it fulfills these requirements. The alternatives to REST include the Simple Object Access Protocol (SOAP), which uses a stricter approach compared to REST, as it is an actual protocol instead of an architecture.

### 2.2. Digitalization of Metrology

The metrology infrastructure is based on standards, mutual trust, and recognition among organizations from around the world. Because of this, there are several national and regional organizations involved in the maintaining of the infrastructure. On the top of the hierarchy are the National Metrology Institutes (NMI), which work together under the International Bureau of Weights and Measures (BIPM) to maintain the metrological standards and guidelines that act as the foundation of the SI unit system [16].

As the need for digitalization in metrology has recently become a key objective for the NMIs and BIPM, there are numerous ongoing research initiatives aiming towards it covering both industrial metrology and legal metrology. In addition to EMPIR SmartCom, European research initiatives such as EURAMET Technical Committee 1448 and GEMIMEG have aimed to advance the development of the DCCs [35,36]. In the United States, National Conference of Standards Laboratories International (NCSLI) 141 MII & Automation Committee is developing the Measurement Information Infrastructure (MII) with a similar aim of making metrological information more available for the purposes of the IoT [37]. For legal metrology, similar digitalization initiatives include the European Metrology Cloud and its spinoff research project AnGeWaNt in Germany [38,39].

#### 2.2.1. Digital SI

The D-SI universal data model has been introduced as a solution for an unambiguous and machine-readable presentation form of metrological data [40,41]. The D-SI format requires that each numerical measurement value be combined with the corresponding unit. This is to prevent the misinterpretation of data due to a lack of or mixing of units. As the name suggests, the D-SI is based on the SI unit system as it is the most commonly used unit system worldwide. However, as other types of units are also commonly used in different domains, the format also supports the inclusion of non-SI units alongside the corresponding SI units. The D-SI also enables including metadata with each individual measurement result, e.g., measurement uncertainty, description of the uncertainty distribution, and timestamps.

#### 2.2.2. Digital Calibration Certificates

Traditionally, calibration results have been documented in calibration certificates, which have conventionally been either printed paper documents or PDF files. Due to this, the calibration information and certificates as a whole have not been available in a machine-readable format, meaning that interpreting the data in calibration management systems or other similar systems has required manual work [13,19,42]. Because of this, the use and value of the calibration certificates have mostly been based on proving the fulfillment of regulations and the compliance of an instrument.

That is why one of the first steps needed in the digitalization of metrology has been defining and developing a digital, machine-readable format for presenting calibration information, i.e., a digital calibration certificate or DCC. For this purpose, different approaches have been proposed [43]. The Swiss NMI Federal Institute of Metrology METAS and the NCSLI have proposed PDF-based formats in which data are embedded in a machine-readable format such as XML [20,23]. XML-based DCC formats have been presented by the German NMI Physikalisch-Technische Bundesanstalt (PTB) and the Association of German Engineers (VDI) [19,21,44]. The benefit of XML as a data format is that its structure can be defined in the form of an XML schema [45,46], which provides the benefits of having several existing technologies, such as cryptographic solutions, available for use in metrological applications, as XML has been in wide use over several years [19]. The presented demonstration uses the DCC format defined by the PTB.

This DCC structure includes sections for the following types of information:

- Administrative information, which is the section for regulated and required information of core interest, such as a unique identifier of the DCC or the information of the calibrated items, customer, and calibration laboratory;
- Calibration results, which is a partly regulated section for the machine-readable measurement results for the calibrated measurands, influence conditions, and other relevant metadata about the calibration procedure such as the used measuring equipment and calibration methods;
- Individual information, which is a non-regulated section for any additional information, such as comments, figures not relevant for the calibration result, individual domain-specific data formats, etc., that are not necessarily machine readable;
- Optional information, which can be considered as a container for metadata about the calibration, such as a human-readable document.

#### 2.2.3. Tracim

To ensure that the measurement data in the DCCs are following the D-SI data model correctly, the data need to be validated. While XML as a file format and the use of XML schemes enables validating the files against a schema, this schema validation does not go as much into detail, e.g., validating that the units are presented in the file using the correct format. That is why a D-SI validation system was developed based on the existing system for Traceability for Computationally Intensive Metrology (TraCIM) [47,48]. Examples of the TraCIM validation process were given in [49].

### 2.3. Data Security in IoT

In many situations, IIoT systems are used in closed environments where the risks for security breaches or cyber attacks are considered to be very minimal. Combined with the rapid development of IoT systems in general, the security aspects of the data exchange outside the data interoperability such as data authenticity, integrity, and confidentiality have often been given a lower priority partly due to a lack of expertise, but also just to spare the costs. As the possibilities of security issues grow when the amount of IoT devices and exchanged data grows and when the benefits of a more open and transparent exchange of data are becoming clearer in many situations, the need for IoT security solutions is growing [50,51]. Technologywise, there are plenty of existing solutions that can also be exploited in IoT systems. However, the scalability requirements set by the large amounts of devices and the volume of data exchanged cause some limitations on the implementation possibilities.

#### 2.3.1. Digital Signatures

Digital signatures are a commonly used cryptographical method for securing files or documents and proving their authenticity and non-repudiation. Applying and validating digital signatures are based on public key cryptography, in which mathematically created digital key pairs consisting of private and public keys are used along with specifically developed hash and signature algorithms, such as the Rives–Shamir–Adleman (RSA) algorithm [52], to compute a fingerprint, also known as a hash, from the original file and from the hash a digital signature for the file that needs to be signed. The keys and algorithms have been defined in a way that if a file is manipulated, the hash of the file will be different. This means that when the receiver wants to validate the signature, it is possible to compute the hash from the signature and compare that to the hash computed from the received document. Only if the hashes are the same, the signature is valid and the document unaltered.

Although the processes for creating and validating digital signatures are relatively simple, the biggest challenges in the use of digital signatures are related to the management of the keys, e.g., ensuring to whom a certain key belongs and what that key is authorized to sign [16]. For this reason, the keys are managed with public key infrastructures (PKIs). In a PKI, the ownership of cryptographic keys is proven with public key certificates, e.g., x.509 certificates, and the trustworthiness of the infrastructure is based on a hierarchy that is comparable to the metrology infrastructure. An example of a well-known and widely used PKI is the infrastructure used for managing the x.509 certificates in the Transport Layer Security (TLS) protocol that is used, e.g., for encrypting communication in the Hypertext Transfer Protocol (Secure) (HTTPS) [53].

The use of digital signatures and their legal validity are dependent on the national or regional laws and regulations. An example of such regulations is the electronic Identification, Authentication and Trust Services (eIDAS) regulations that are in use in the European Single Market [54].

#### 2.3.2. Distributed Ledgers

Although digital signatures are very effective at securing the data authenticity and integrity, the security of the system can be further improved with distributed ledger technologies (DLTs), often referred to as blockchains, which are trusted, shared, and append-only databases [55]. In short, distributed ledgers have two defining features:

- The ledger database is distributed, meaning that there exists up to thousands of copies of the database. The database is maintained by nodes that compute the transactions according to an agreed upon consensus protocol;
- The transactions cannot be changed or removed afterwards once they have been entered into the ledger. This is achieved by using cryptographic identifiers to chain the transactions, which are packaged into blocks, to each other, hence the common name blockchain.

The benefits derived from these features become very apparent in business environments such as logistics, where the traceability, origin, and non-repudiation of both the transported goods and related information are essential and the parties involved may not know and trust each other by default. The main benefits of the distributed ledgers in this kind of an environment are the trust achieved through the databases being distributed by definition and transparency, which combined lead to the possibilities to reduce costs. That is why DLTs are being investigated and implemented in various applications in logistics chain management [56,57,58,59]. A notable example of a commercial DLT implementation is TradeLens (https://www.tradelens.com/, accessed on 5 September 2021), which is an open and neutral supply chain platform developed by IBM and GTD Solution Inc. in collaboration with Maersk.

## 3. Materials and Methods

**Figure 1:**
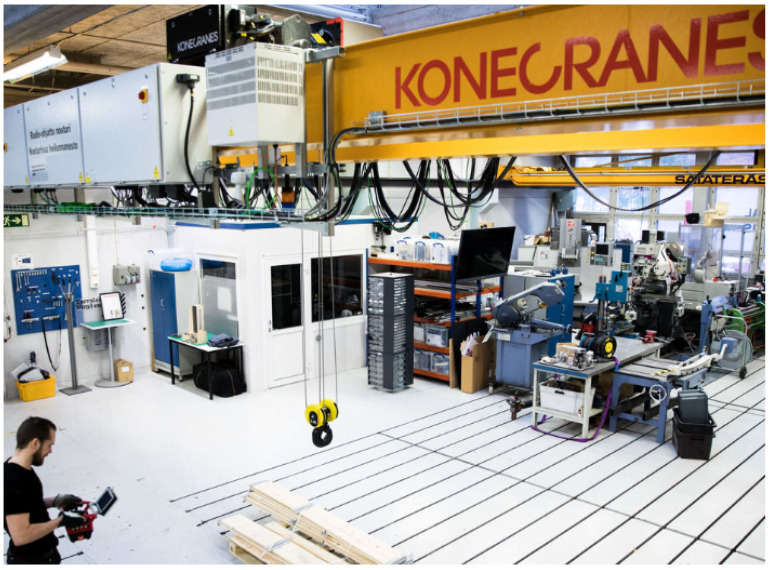
The smart overhead crane at the Aalto Industrial Internet Campus.

The development of the IoT has brought up new kinds of needs for expertise in the industry. Because of this, the Aalto Industrial Internet Campus (https://www.aalto.fi/en/aiic, accessed on 20 August 2021) (AIIC) was founded to support the multidisciplinary education of mechatronics and the IoT. At the center of the AIIC is the smart overhead crane, Ilmatar, which provides the cyber–physical platform for research, innovation, and education activities [60]. Ilmatar is a Konecranes CXT Crane that has a maximum lifting capacity of 3.2 tonnes. The crane system consists of three subsystems: the hoist, the trolley, and the bridge, which also act as the three-dimensional movement axes of the crane. The crane is shown in Figure 1, and the features and corresponding sensors of the crane subsystems are presented in Table 1. In addition to the basic operation of the crane, the sensors enable numerous smart features (https://www.konecranes.com/sites/default/files/download/konecranes_brochure_smart_features_en_2015.pdf, accessed on 20 August 2021) such as active sway control, target positioning, and predicting of the hoist brake system maintenance interval. These more advanced features are dependent on a larger amount of data and more in-depth data analysis than the basic operational features of the crane; thus, they are also more dependent on the data quality and trustworthiness.

The demonstrator system focuses on the features and measurement systems on the crane that are used to collect the data that are the most important for the harbor and carrier operators in the logistics chain, i.e., the crane position and load measurements. For position measurements, the focus is specifically on the laser distance sensors used to measure the position of the crane trolley and bridge. Due to the sensors being identical, a single sensor was calibrated and the calibration results were used to create a DCC. The calibration of the sensor was performed at VTT MIKES in Otaniemi, Finland. The DCC of the load was created following a similar structure as the DCC of the laser sensor because a similarly precise calibration of the load measurement system was not possible due to the system being an integral part of the crane.

### 3.1. Tools and Software

The demonstrator functionalities were implemented with APIs specific to each functionality and a Main API for running the other APIs. The demonstrator includes the following features and APIs:

- Main API;
- OPC UA client;
- DCC API;
- eIDAS signing service;
- Database API;
- SQL database;
- User interface (UI).

The components of the demonstrator were programmed using Python (Main API, OPC UA Client, Database API), Java (signing service), and JavaScript (DCC API and UI). The source code is available at the Ceracrane gitlab repository (https://gitlab.com/aalto-smartcom/ceracrane, accessed on 30 August 2021). Due to the system having several software components, the individual services were packaged into Docker containers to simplify the management of the software code and dependencies in the development phase and ensuring the reliability of the services. A Docker container image is a lightweight, standalone, executable package of software that includes everything needed to run an application: code, runtime, system tools, system libraries, and settings [61]. The defining and running of the containers were implemented using Docker Compose (https://docs.docker.com/compose/, accessed on 10 September 2021).

Due to renovation work at the AIIC facility, a separate mockup OPC UA server was created to act as a simulation of the crane OPC UA server to allow the use and testing of the demonstrator even when the crane is offline.

### 3.2. Design Architecture and Implementation

**Figure 2:**
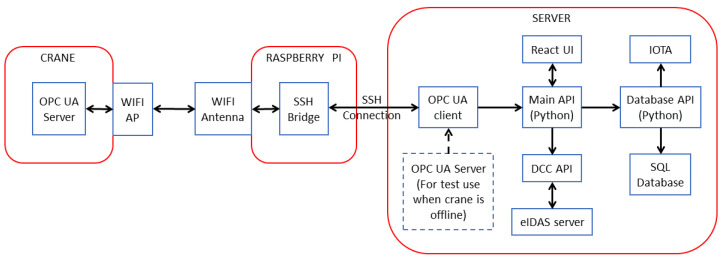
The design architecture of the demonstrator system.

The design architecture of the system is presented in Figure 2.

#### Data Exchange

The data collected by the sensors in the crane are obtained from the crane OPC UA interface. For this purpose, a universal PC, in this case a Raspberry Pi, was deployed in the research facility where the crane is located as a WiFi contact point providing a Secure Shell (SSH) tunnel, i.e., a secure channel through the open network between the crane OPC UA server and OPC UA client on the server running the demonstrator.

The measurement files are eventually stored in a SQL database, from which the users can search and view the measurements of specific containers using the UI.

### 3.3. Data Formats

The measurement data sample obtained from the crane OPC UA interface are saved in measurement files that use a structure defined by an XML schema. The XML file structure includes three parts:

- Measurement metadata. In the measurement file format, metadata refer to the name or identifier of the device or system from which the data are obtained, which in this case is the crane, a timestamp including the complete date plus hours, minutes, and seconds (YYYY-MM-DDThh:mm:ssTZD) as defined in Section 5.4 of the ISO 8601 standard [62], and an identifier for the container that is being lifted and measured;
- Measurement results. The measurement result section of the schema includes elements for the gross weight, tared weight, bridge position, hoist position, and trolley position obtained from the crane OPC UA interface. The results are structured using the D-SI schema Version 1.3.1 (https://www.ptb.de/si/v1.3.1/SI_Format.xsd, accessed on 20 August 2021);
- XML signature. The signature format used in the measurement XML structure is discussed in Section 3.4.

An example of the measurement data and metadata format was presented in [63].

DCCs are used in the demonstrator to prove that the sensors have been calibrated appropriately and that the measurements are traceable, thus verifying the data quality and trustworthiness. The DCCs of the measurement instruments were created according to the DCC schema Version 3.0.0-rc.2 (https://www.ptb.de/dcc/v3.0.0-rc.2/dcc.xsd, accessed on 20 August 2021).

### 3.4. Data Security

Data security solutions were implemented in the demonstrator to protect the system from data altering attacks. The main criteria for the security solutions were:

1. Preventing that a measurement could be added, removed, or altered without the users being notified;
2. The ability to validate the authenticity of the DCCs associated with the measurement results.

Based on these requirements, the security solution for the system was chosen to include digital signatures and the possibility to validate them to ensure that data could not be altered or fake data from a third party could not be used, as well as a blockchain implementation to further improve the possibilities to validate the authenticity and integrity of the data and ensure the non-repudiation of the data.

During the measurement file creation process, the files were digitally signed to prove the authenticity and integrity of the measurement values. The signature service used in the demonstrator to sign the measurement files was developed based on technologies that fulfill the eIDAS regulation. For XML signatures, the eIDAS specifies XML Advanced Electronic Signatures (XAdES) [64], which introduces six additional forms to the XML digital signature standard (XML-dsig) [65]. The implementation of the signing service was based on examples developed as a part of eSignature, which provides a set of free standards, tools, and services developed to help accelerate the use of legally valid electronic signatures in the Member States of the European Single Market as a part of the Connecting Europe Facility (CEF) program [66].

For the blockchain solution, the system uses an IOTA-based (https://www.iota.org/get-started/what-is-iota, accessed on 20 September 2021) blockchain implementation to secure the non-repudiation of the data, e.g., by preventing an attacker from deleting or replacing measurement files without other users noticing it. The data structure behind IOTA is known as IOTA Tangle (https://blog.iota.org/the-tangle-an-illustrated-introduction-4d5eae6fe8d4/, accessed on 18 January 2021), and it is well suited for IoT applications. For the IOTA transaction, a message, i.e., a collection of data of a single measurement event that are stored, is created using JSON. The transaction message contains the following information:

- Crane/measurement system identification;
- Container identification;
- Measurement values related to the container;
- Timestamp;
- XML string of the measurement file;
- Fingerprint of the signed measurement XML;
- Reference to the DCC, i.e., a cryptographic identifier [63].

The algorithm used for the IOTA transaction creation and validation is presented in Appendix A. Once the IOTA transaction is complete, the same information accompanied by the IOTA transaction hash is stored to the SQL database.

### 3.5. User Interface

The user interface of the demonstrator was developed using React Native (https://reactnative.dev/docs/getting-started, accessed on 10 September 2021) and JavaScript. The UI has two main views:

- Crane operator view for creating measurements;
- Container measurement search and validation view.

In both views, the user can inspect the information of the measurement devices including the possibility to validate the DCCs of the devices. The features of the UI were presented in more detail in [63].

## 4. Results

### 4.1. Creation of a Measurement in the Crane Operator View

In the operator view, the user can input the container identification and click “create a measurement” to start the measurement process. Before the user can create a measurement, the Main API must first connect to the crane’s OPC UA server. This setup phase has the following steps:

1. The OPC UA client connects to the crane’s OPC UA server via the SSH tunnel;
2. Data from the sensors are fetched to the Main API;
3. Once the data have been retrieved, the user can now start the process for creating a measurement.

After the connection process, the user can start the measurement process, which consists of the following steps:

1. The measurement process is started by the Main API;
2. The Main API collects the measurement data from the crane OPC UA over the SSH tunnel;
3. The Main API creates the XML measurement file, which is then sent to the DCC API;
4. The DCC API relays the file to the eIDAS server, and the file is signed;
5. The signed file is sent to the Database API, where a digital fingerprint, i.e., a hash of the file, is computed;
6. The file is sent to IOTA. The algorithm used for the IOTA transaction is presented in Appendix A.1;
7. The IOTA transaction hash is attached to the information, which is then stored to the SQL database.

**Figure 3:**
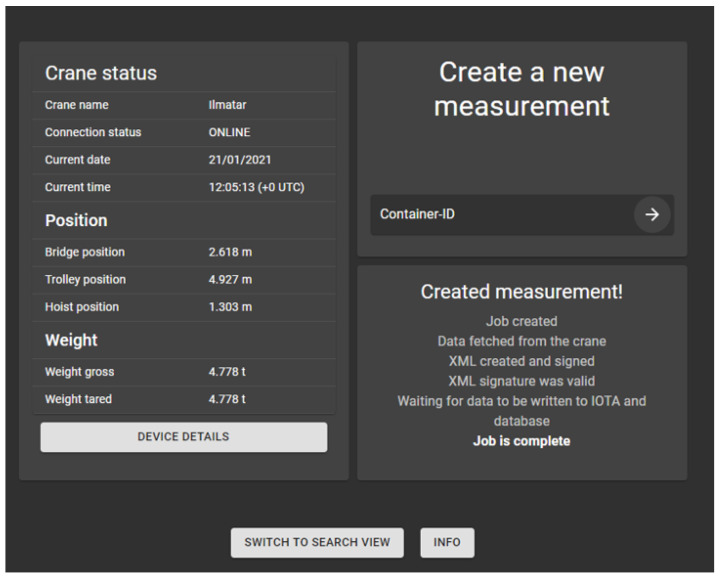
The operator view of the UI showing that a measurement has been successfully sent to IOTA and saved into the database.

Figure 3 shows the UI after a successful measurement event.

### 4.2. Container Measurement Search and Validation

In the search view, the user can inspect all measurements specific to a particular container that have been saved in the database by inputting the container identification. All the measurements are then validated automatically. The search and validation process goes as follows:

1. The Main API receives a request from the UI;
2. The request is forwarded to the Database API;
3. Te Database API retrieves the information from the SQL database and validates the transaction from IOTA. The algorithm that is used in the IOTA validation is presented in Appendix A.2;
4. The measurement and validity information are returned to the Main API, which sends them to the React UI.

**Figure 4:**
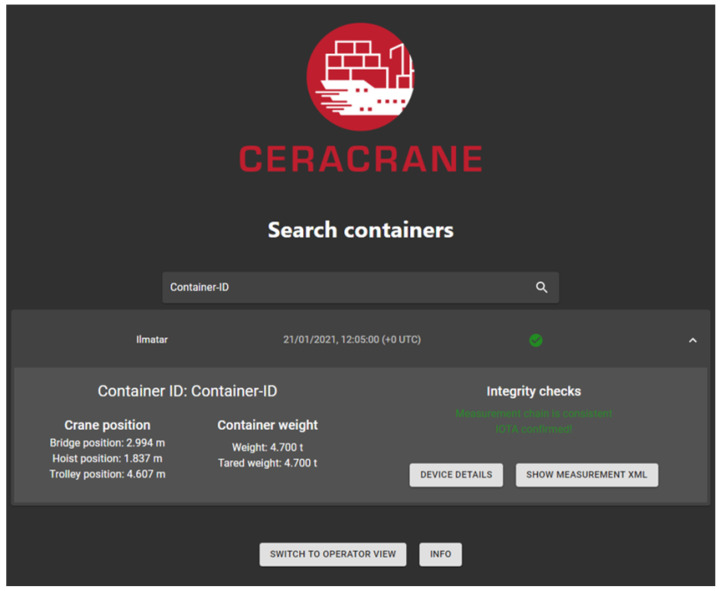
The search view of the UI showing a validated measurement.

Figure 4 shows an example of a validated measurement event.

### 4.3. Measurement Device Information

**Figure 5:**
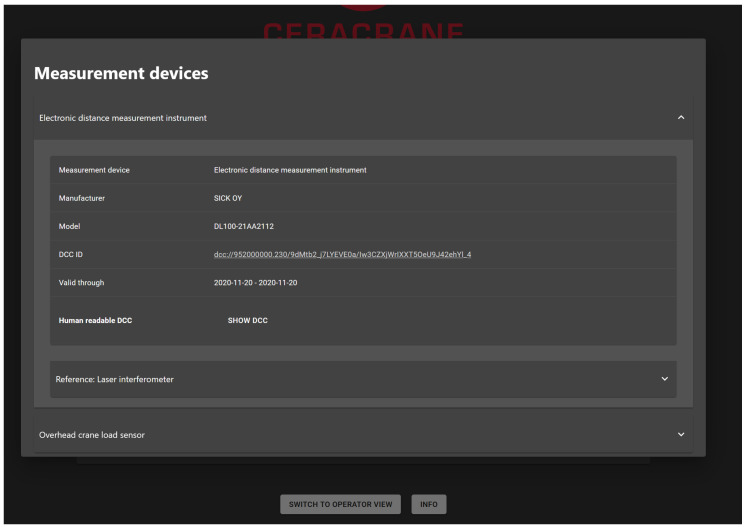
The UI showing the information of the measurement devices in the device details view.

**Figure 6:**
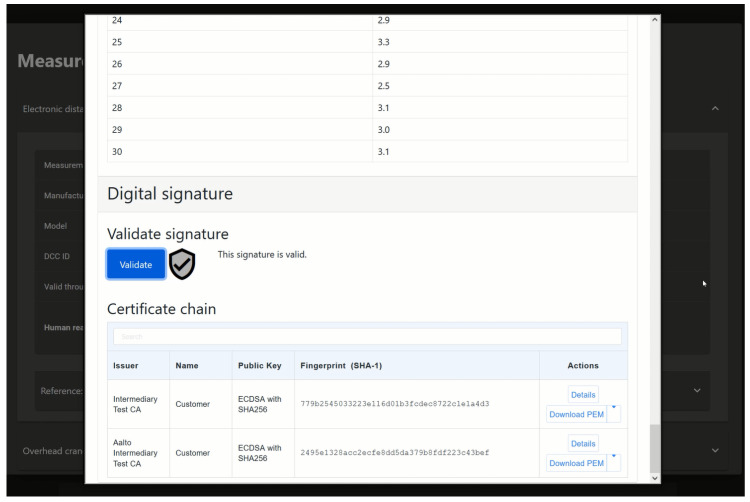
The UI showing that the digital signature of a DCC is valid. Below the validation tool is information about the public keys of the authority that created the signature and the issuer of that public key.

Information about the measurement devices used to collect the data can be viewed in both views of the UI. This includes the possibility to inspect the DCCs of the measurement instruments used to collect the data. Figure 5 shows how the device details view is displayed in the UI. The user can view the DCCs in a human-readable format, which also allows the user to validate the digital signatures and shows the information of the public key that was used to create the signature and the PKI of which the key in question is a part. Figure 6 shows how the information and validation of the DCC are presented in the UI.

### 4.4. Testing and Validation

To validate the functionalities of the system, tests were performed to ensure that misuse or attacks can be identified by the system and the users are notified. The test scenarios are presented in Table 2. The IOTA validation method is described in detail in Appendix A. The system and the tests were run locally using a mock-up PostgreSQL (https://www.postgresql.org/about/, accessed on 18 January 2021) database in which test data were written and read using a Python code that allowed manually replicating the operations of the Database API. The duration of each database validation test varied as they were dependent on IOTA Tangle. The test scenarios had no significant effect on the duration of the validation of the database as the applied validation process was the same. The validation of the digital signatures of the DCCs was quicker as it was only dependent on the processing capabilities of the hardware running the system locally and the signing server, which in this case were a workstation laptop and a conventional office server.

The tests indicated that the system handled the different scenarios as intended, fulfilling the security requirements and design goals defined for it. The durations of the validation processes were considered to be sufficiently quick as optimizing the performance of the system was not a top priority for the demonstration.

## 5. Discussion

By combining the principles and technologies based on metrology and data security, the presented system offers a comprehensive protection for the data exchange between the users and storing of the data. The use of D-SI minimizes the possibilities for errors due to the wrong interpretation of the data as the measurement values are always presented with the corresponding SI units in a machine-readable format. The inclusion of the DCCs in the system allows the users to check that the devices used to collect the data have been maintained appropriately, and thus, the data can be considered to be sufficiently accurate. The digital signatures and IOTA ensure that the users can trust that the data are originally from the source from where they are supposed to be, they have not been manipulated, and no counterfeited measurements have been added or real measurements removed without the validation system notifying them.

When implementing a digital solution of any sort, it is always essential to understand the data security risks and needs the implementation brings with it. For example, in the use case presented, if just the DCCs were used in the system to enhance the data trustworthiness by proving that the devices have been calibrated and maintained in accordance with the requirements, the system would have vulnerabilities that would significantly compromise the benefits and, in the worst case, cause significant issues. An important thing to keep in mind in the implementation of a data security solution for any kind of a system is that the cost efficiency of the system is dependent on the risk assessment. Inadequate or excessive solutions can lead to scenarios where the system is either too vulnerable to attacks or unnecessarily costly to maintain.

### 5.1. Opportunities in the Digitalization of Metrology

The digitalization of metrology can provide significant benefits in understanding and considering data quality in IoT applications. Without the reliable indication of the traceability or integrity of the data, the value of the data in any application is limited compared to what it could be. Trustworthy and interoperable data provide many new possibilities for the use of these data, e.g., through the more open exchange of data between parties [55,67]. More open availability and transparency of calibration information could also benefit uncertainty analysis, improving the data quality and value further, as presented in [17,18].

#### 5.1.1. Dynamic Uncertainty Information and Metadata

Currently, the majority of IoT systems do not include a means for assessing or proving data trustfulness, as systems based on networks of low-cost IoT sensors typically lack traceability to measurement standards and thereby to the SI units [12]. Additionally, the recent trends for decentralization in IoT sensor networks are somewhat challenging from a metrological point of view, as decentralization can be considered to be conflicting with the hierarchy on which the metrology infrastructure is based [13].

Calibrating the sensors in the networks and having the calibration information available for assessing the measurement uncertainty dynamically for each individual measurement point would provide significant benefits, e.g., for cyber–physical systems, where measurement data are used for simulating the state and behavior of physical objects [8,30,42]. Having the measurement uncertainty available would allow taking it better into account in the simulations.

#### 5.1.2. Online Compensations

The availability of calibration information in an easily processable format could enable more specific understanding of the conditions where the measurements are conducted. In a production environment, this would allow more advanced methods for compensating the measurement errors and corresponding uncertainties. For example, temperature variations during machining processes cause fluctuations in the dimensions of large workpieces. With a large amount of data available, the behavior of the measurement instruments and corresponding effects on the measurement results can be identified and appropriately compensated.

#### 5.1.3. More Open Exchange of Data

One of the limitations hindering more open data exchange between different organizations is the lack of verification of the quality and trustworthiness of the data. This problem of course falls directly within the scope of metrology, and by nature, it is no different from the past challenges that led to the formation of the metrology infrastructure in it current form. Digitally available metrological data used to indicate the quality and trustworthiness of data would offer a solution to this lack of trust between the parties and enable new uses for data that may not have been previously considered possible.

### 5.2. Remaining Challenges

Although the research initiatives such as SmartCom are taking the world of metrology in the right direction towards digitalization, there are still some significant challenges remaining before the digitalization of metrology reaches the state when all of the potential benefits become available for implementation in industrial-grade applications. These challenges mostly arise from the strong establishment of the current practices and how the metrology domains and infrastructure are accustomed to be and work. For example, the perception of the significance of signatures in calibration certificates and other similar documents can often lead to misconceptions relating to the purpose of and need for digital signatures as they can be considered to be equal, although the security provided by the digital signatures goes far beyond handwritten signatures.

#### Harmonization of Data Formats and Procedures

In the case of SmartCom, the DCC and D-SI have been introduced to be the basis for the unambiguous and secure exchange of metrological data. This can only be achieved if they are widely accepted and proven to be sufficiently comprehensive. As metrology is heavily based on the international cooperation of organizations ranging from world-wide corporations to small service providers, the capabilities for the uptake of new formats and technologies can vary significantly.

One of the challenges in achieving the critical number of industries behind new technologies is the ability to take domain-specific requirements into account. These requirements can be based on, e.g., legislation or quantity-specific physical phenomena. Importantly, these requirements should not be mixed with accustomed ways of doing and presenting things. In metrology-related applications, the requirements and current practices can vary greatly between different domains and industries as the methodologies and the types of instruments used to measure different physical quantities vary significantly. In that regard, one of the aims of the digital transformation would also be harmonizing procedures, standards, and guidelines where possible.

In its current format, the DCC schema provides a basis for development, but there are still numerous steps that still need to be taken before the domain-specific requirements can be considered to be fulfilled. That is why close the collaboration of the leading metrology organizations and the relevant industries is essential for driving the work onward and growing the community around it. The examples of communities such as the OPC Foundation and NAMUR have shown how technologies can relatively efficiently achieve the status as a de facto standard and eventually an industry-wide standard, once the critical number of members in the user and developer community is achieved.

### 5.3. Development towards Digital Metrology Infrastructure

The development in the digitalization of metrology is inevitably going to lead to major changes in the metrology infrastructure. New requirements based on the new technologies will lead to a need for the key metrology organizations to adapt into new roles as, e.g., the use of digital signatures will be necessary to maintain the mutual trust of the current infrastructure in the transition towards the digital world [15]. Fortunately, the fact that metrology has been slow in digitalization means that there are also many examples of societal or industrial applications where similar steps have already been taken.

## 6. Conclusions

In this paper, we presented a method for using previously unavailable and unused metrological information as metadata to enhance the interpretation of measurement data. To further improve the trustworthiness and usability of the data, methods for using digital signature and DLT-based solutions to ensure data security were also proposed. The use of the digital metrological data and the proposed security solutions were demonstrated in an industrial application by developing a system integrated with a smart overhead crane located at Aalto University’s AIIC facility. The operation of the system and its security solutions were successfully tested and validated against various data-tampering attacks.

The availability of the metrological data in a machine-readable and processable format enables new possibilities in data usage as the true meaning and context of each measurement and corresponding value can be understood in more detail. The metadata can be used to assess the quality and thus the value of the data for the analytical purposes that are typical in IoT systems. However, without the means to indicate the authenticity and integrity of the data, this value is lost. For this reason, data security should be a top priority in the digitalization of metrology, as in any other domain.

Due to the complexity of the metrology infrastructure, there are still plenty of open research questions to be answered and necessary changes in the current practices and attitudes before the full benefits of metrology will be exploitable in the digital world. However, with a laborious transformation also comes great possibilities to improve the overall value and usability of measurement data in all sectors of industry and society. The collective efforts around the world for the digitalization of metrology have established a solid basis on which the following research initiatives can be built. Examples from other similar efforts have shown that with a large enough community with industrial actors willing to become forerunners, standardization can be achieved even on a global level.

## Figures and Tables

**Table 1:** Features and corresponding sensors of the smart overhead crane subsystems.

Subsystem	Feature	Corresponding Sensor (s)
Hoist	Speed control and position measurement	Konecranes NM701NR3 encoders integrated in the hoist motors and DynAHoist Vector II variable-frequency drive
	Load measurement and overload protection	Load-cell-type load break sensor and ControlPro unit
	Rope angle measurement	Inclinometer at the static end of the hoisting ropes
	Brake monitoring unit	Current sensor and the sensors used for hoist controls
Trolley	Speed control	Konecranes NM701NR3 encoders integrated in the hoist motors and DynAC Vector II variable-frequency drive
	Position measurement	SICK DL100-21AA2112 laser distance sensors
Bridge	Speed control	Konecranes NM701NR3 encoders integrated in the hoist motors and DynAC Vector II variable-frequency drive
	Position measurement	SICK DL100-21AA2112 laser distance sensors
	Anti-collision	Same sensors as for speed control and position measurement

**Table 2:** Test scenarios used for the validation of the demonstrator. The exact validation durations are dependent on the used hardware. As anticipated, in the case of the demonstrator, all tests were evaluated as passed.

Test Scenario	Outcome	Validation Durations	Evaluation
DCCs:			
A DCC of the crane’s sensor is altered in the database.	The user (either the crane operator or other user) can validate the DCC from the device details section in the crane operator view or search view. If the DCC has been changed, the signature validation fails.	1–5 s	Pass/fail
A digitally signed fake DCC of the crane’s sensor is used in the system.	In addition to the signature validation, the user can see by whom or which organization the DCC has been signed, so even a real signature created by a third party can be identified.	Instantaneous (the information is included in the DCC XML from which it is displayed in the UI).	Pass/fail
Database:			
A measurement in the database is altered.	Due to the alteration, the measurement file no longer matches the original XML string of the measurement that is included in the IOTA transaction. The system informs the user that the IOTA transaction validation is invalid.	5–20 s	Pass/fail
Addition of a fake measurement to the database.	The number of measurements in the database and IOTA do not match. The system informs the user that the IOTA validation is invalid.	5–20 s	Pass/fail
Removal of a measurement from the database.	The number of the measurements in the database and IOTA do not match. The system informs the user that the IOTA transaction validation is invalid.	5–20 s	Pass/fail
Replacement of a measurement in the database with a fake measurement.	The transaction tag of the replacement measurement does not match the tag of the replaced measurement. The system informs the user that the IOTA transaction validation is invalid.	5–20 s	Pass/fail
Addition of an IOTA transaction that has a correct transaction tag, but the message is fake.	The decrypted message of the added transaction does not begin with a prespecified tag so the transaction is not used for confirming measurements in the database. The system informs the user that the IOTA transaction validation is invalid.	5–20 s	Pass/fail

## Data Availability

Publicly available datasets were analyzed in this study. This data can be found here: https://gitlab.com/aalto-smartcom/ceracrane (accessed on 30 August 2021).

## Abbreviations

The following abbreviations are used in this manuscript:

AIIC	Aalto Industrial Internet Campus
API	Application programming interface
BIPM	International Bureau of Weights and Measures (in French: Bureau international des poids et mesures)
CEF	Connecting Europe Facility
DCC	Digital calibration certificate
DLT	Distributed ledger technology
D-SI	Digital SI
eIDAS	Electronic Identification Authentication and Trust Services
HTTP(S)	Hypertext Transfer Protocol (Secure)
(I)IOT	(Industrial) Internet of Things
IMO	International Maritime Organization
JSON	JavaScript Object Notation
MII	Measurement Information Infrastructure
MQTT	Message Queuing Telemetry Transport
NOA	NAMUR Open Architecture
NMI	National Metrology Institute
NCSLI	National Conference of Standards Laboratories International
OPC UA	Open platforms Communication Unified Architecture
PDF	Portable Document Format
PTB	German NMI, Physikalisch-Technische Bundesanstallt
PKI	Public key infrastructure
REST	Representational State Transfer
RSA	Rives–Shamir–Adleman signature algorithm
SOAP	Simple Object Access Protocol
SOLAS	Safety of Lives at Sea Convention
SSH	Secure Shell Protocol
SQL	Structured Query Language
TLS	Transport Layer Security Protocol
TraCIM	Traceability for computationally intensive metrology
UI	User interface
VDI	Association of German Engineers (in German: Verein Deutscher Ingenieure)
XAdES	XML Advanced Electronic Signature
XML	Extendable Markup Language
XML-dsig	XML Digital Signature Standard

## Appendix A. Algorithm for Validating a Measurement in the Database against IOTA

When reading measurement data from the database through the REST API, the data are validated against IOTA to confirm they have not been modified since storing them. This section describes how the validation algorithm works. The algorithm relies on two assumptions:

1. The key used for the Fernet cipher (https://cryptography.io/en/latest/fernet/, accessed on 18 January 2021) is stored securely;
2. Data written to IOTA are immutable.

### Appendix A.1. Creating an IOTA Transaction

When data are stored, a JSON string is created from the measurement data:

(A1) j=JSON(measurement_data)

The JSON string is hashed with SHA-256:

(A2) h=SHA-256(j)

The SHA-256 hash is prepended with a prespecified tag, which is kept secret, in this example “CERA”:

(A3) p=‘CERA’+h

The resulting string, *p*, is encrypted with the Fernet cipher, which uses the Advanced Encryption Standard (AES) in cipher block chaining (CBC) mode:

(A4) c=fernet_enc(key,p)

An IOTA transaction is created. The tag of the transaction is a 6 bit Blake2B (https://datatracker.ietf.org/doc/html/rfc7693, accessed on 18 January 2021) hash of the container ID. The message of the transaction is the ciphertext created in the previous step, *c*. The transaction is stored in Tangle, and the measurement data accompanied by the IOTA’s transaction hash are stored in the database.

### Appendix A.2. Confirming an IOTA Transaction

When data are retrieved, their integrity is checked as follows. The container ID whose measurements are to be read is passed as a parameter to the REST API. The six-byte Blake2B hash is calculated from the container ID. Tangle is searched for all transaction having the hash as their tag.

The message, *m*, of each found transaction is decrypted with the Fernet cipher:

(A5) q=fernet_dec(key,m)

The transactions are validated by checking their decrypted message; *q* begins with the prespecified tag, CERA:

(A6) q[0:4]=‘CERA’

A set *I* is formed of the valid transaction messages:

(A7) I={q:q[0:4]=‘CERA’}

A set *D* of all the measurements in the database with the given container ID is formed. The sets *I* and *D* must equal in size. If not, none of the measurements in the database are confirmed. If the sizes of the sets are equal, an SHA-256 hash, *h*′, is calculated for each measurement in the database and prepended with the prespecified tag,

(A8) $$r=‘\mathrm{CERA}’+h^{\prime }.$$

A measurement in the database is confirmed if the resulting string is found in the set of valid IOTA transactions:

(A9) I={r,…}

Otherwise, the measurement is marked as unconfirmed. A list of all the measurements in the database with the given container ID is returned from the API. The list indicates for each measurement whether it was confirmed or not.

The algorithm counters the following attacks:

- Modifying a record: *r*, not found in the valid IOTA transactions, *I*, when reading from the database;
- Removing a record: The number of records in IOTA, |*I*|, and database, |*D*|, do not match;
- Adding a record: The number of records in IOTA, |*I*|, and database, |*D*|, do not match;
- Removing a record and adding a new one: *r*, of the new record not found in valid IOTA transactions *I*;
- Adding an IOTA transaction with the correct IOTA transaction tag, but nonsensical message: Decrypted message of the added transaction does not begin with the prespecified tag,
  (A10) (q[0:4]!=‘CERA’),
  so the transaction is not used for confirming measurements in the database.
